# Traceability in Patient Healthcare through the Integration of RFID Technology in an ICU in a Hospital

**DOI:** 10.3390/s18051627

**Published:** 2018-05-19

**Authors:** María Martínez Pérez, Carlos Dafonte, Ángel Gómez

**Affiliations:** CITIC—Department of Computer Science, University of A Coruña, Campus de Elviña s/n, 15071 A Coruña, Spain; dafonte@udc.es (C.D.); angel.gomez@udc.es (Á.G.)

**Keywords:** NFC, traceability, adverse effects, ICU

## Abstract

Patient safety is a principal concern for health professionals in the care process and it is, therefore, necessary to provide information management systems to each unit of the hospital, capable of tracking patients and medication to reduce the occurrence of adverse events and therefore increase the quality of care received by patients during their stay in hospital. This work presents a tool for the Intensive Care Unit (ICU), a key service with special characteristics, which computerises and tracks admissions, care plans, vital monitoring, the prescription and medication administration process for patients in this service. To achieve this, it is essential that innovative and cutting-edge technologies are implemented such as Near Field Communication (NFC) technology which is now being implemented in diverse environments bringing a range of benefits to the tasks for which it is employed.

## 1. Introduction

A principal concern for health professionals within the hospital environment is to reduce the occurrence of adverse events [1,2,3,4,5,6] in the care process of the patient. An adverse event is understood as any complication that arises during the stay of the patient in hospital and which is not directly related to the base illness or reason for admission. These incidents can have grave consequences for the patient and family, generating elevated costs and undermine confidence in the health system, ultimately undermining institutions and the health professional. Patient safety is now considered a priority by leading health organisations such as The World Health Organisation and international bodies such as the EU, the Council of Europe, health authorities, professional societies and patient organisations.

There are many international studies [7,8,9,10,11,12] dedicated to the study of adverse events in health systems, the origin and the improvements that can be undertaken to improve the tasks involved in clinical practice. This is a concern in Spain where economic, technological and human resources have been employed to attempt to achieve a zero rate of occurrence of this type of problem within the healthcare environment. These national studies [13,14,15,16,17] have revealed important data and have focussed on a range of healthcare areas with the analysis of diverse parameters. The Ministry of Health, Social Services and Equality, which has overall responsibility for the improvement of quality in the system and regulated by the 16/2003 legislation for the cohesion and quality of the National Health Service, considers patient safety as the key component of quality and is situated at the centre of health policy.

This is reflected in the 8th Quality Assurance Plan of the National Health Plan, the objective of which is to improve safety in national health centres through a range of initiatives among which are: to promote and develop the culture and awareness of safety among professionals and patients; to design and establish information and notification systems of adverse events for learning purposes; and implement recommended safety practice in national health centres. This strategy is based on the recommendations of the World Alliance for the Safety of Patients (related to the WHO) and other international bodies [18]. The concept of traceability can provide many benefits to these processes.

Traceability is understood as identifying all the information related to a product from its origin to delivery and/or consumption. In the health service environment, this can be translated as the precise identification of the patient, medication and administered patient/medication relation. This all reduces the incidence of adverse events thereby increasing the safety of the patient. At the same time, these initiatives contribute significantly to the sustainability of both the public and private healthcare systems given that an adverse event normally provokes a prolonged stay of the patient in the hospital and/or the administration of extra medicine to remedy adverse effects. In addition, legal costs can emerge to clarify and/or indemnify the patient as a result of an adverse incident.

The process for obtaining traceability in the healthcare environment is focused on the unambiguous identification of the patient, medication (name, lot number, expiry date, indications for administration and storage, etc.) and the administered patient/medication binomial. To complete the tracking of patients and medication in real time, it is necessary to unequivocally identify the health personnel in charge of each task in the care process and their location.

Through data analysis of the tracking register, one can measure the efficiency of each one of the services, detect bottlenecks so that actions can be undertaken to improve weak points detected in the process under analysis. Innovative and cutting-edge technologies such as RFID (Radio Frequency Identification) are now being successfully integrated into healthcare environments [19,20,21,22,23] and can facilitate the accomplishment of the previously described tasks [24,25,26,27].

This work centres on the intensive care unit (ICU) of the Virxe da Xunqueira de Cee hospital complex [28], which serves the entire area of Costa da Morte [29] and represents a point of reference for the the care process of the patient. The system that has been implemented has arisen from the needs demonstrated by the clinical personnel of this unit.

The ICU is particularly specialised given that its patients suffer from serious illnesses who have to be attended to with promptness and capacity [30]. Prior to the onset of this work, the processes involved in the care of patients could only be recorded on paper. The possibility of automating these through innovative technologies, to minimise the occurrence of adverse events and thereby improve safety and efficiency, is very attractive indeed. These parameters contribute to the quality of care received by the patient; in fact, legislation does exist with recommendations covering the care process and administration of medication to patients in ICU and is outlined as follows:

- Standardise communication between professionals and different units during changes of shifts and at the time of transfer of a patient from one unit or specialist treatment area
- Pay special attention to high-risk medication managed in the ICU: storage conditions will be reviewed and periodically updated directives will assure the standardisation of their preparation and administration (the required concentrations of solutions for infusion, administration schedules, criteria for the use of infusion pumps, etc.).
- Promote the implementation of new information and communications technologies in the ICU especially the availability of computerised medical records and assisted electronic prescription, integrated in the medical record of the patient and connected with the laboratory, with alerts for medicine-medicine and medication-condition interactions, maximum dosage and dose adjustment, allergies and inappropriate medicines for older people.
- Up-to-date protocols will be available for treatment and/or guides to the use of medication based on the evidence from the principal illnesses treated in the ICU so as to standardise the prescription and the tracking of the effectiveness and safety of the treatment. These protocols will consider the adequacy of the dosage in older patients and recommend systematic adjustments to the dose of a medicine at the time of prescription, taking into account age, the renal and liver function of the patient.

The implemented system contributes to the fulfilment of these recommendations through the integration of NFC technology. NFC is a specific protocol for one of the operating frequencies (HF) of RFID technology.

It is important to highlight that the health professionals of the centre have participated at all stages in the lifecycle of the project and have adjusted the functionalities and usability of the work to their requirements in daily clinical practice in the ICU.

## 2. Objectives

The aim of this work is the integration of RFID technology into the tasks carried out by health professionals in a hospital intensive care unit. The work intends to achieve, more specifically, the following objectives:

- The development of a tool to manage the care process of patients admitted to an ICU: registration of patient medical data; the electronic prescription and administration of the medication; monitoring and alerts (if necessary) for vital signs and finally the doctor’s discharge of the patient.
- The precise identification of health personnel, the patient, medication as well as the binomial patient/medication (prescribed or administered). Each person involved should have the unique RFID tag which offers the relevant information during the care process.
- The localisation, almost in real time, of patients who have been administered a specific lot of medication so that possible pharmaceutical alerts can be quickly managed.
- Facilitate rapid and efficient computerised information among health personnel relating to, for example: the transfer of patients from one unit or other area within the service, care plans, detected alerts, medication for administration, pending tasks, etc.

The objectives described above have been carried out through the implementation of an RFID system to obtain traceability of patients, medication and health personnel and is described in the following sections. The active participation of the health personnel (its end users) facilitated the achievement of the necessary functionalities and usability requirements, reducing the learning and training time required from the staff and assuring the success of the project in daily clinical practice.

## 3. Development

### 3.1. Analysis

The first stage of the project focuses on the functioning of the care process of the patients in the ICU. The objective is to identify those points where adverse events are possible and, as such, ideally suitable for the implementation of RFID. The developed RFID system is made up of distinctive subsystems devised to obtain the traceability of patients, medication and the health professionals involved. With this, actions can be undertaken to improve those weak points in which human errors can occur and, in so doing, increase the safety of the patient by reducing the occurrence of adverse events.

The methodology for accessing the knowledge of the health professionals involves holding periodic meetings for all those involved (doctors, nursing staff, nursing assistants, ancillary staff). Having a multidisciplinary team allows for the inclusion of different points of view from the future users of the new system. This participation strengthens the possibility of achieving the tasks within the existing and established protocols. A major advantage is that it reduces learning time and the possible resistance to change on the part of the personnel involved.

Each meeting gives rise to documentation in the form of a diagram which represents the flow of work in the process under analysis which will be refined in a subsequent meeting or finally approved by a team of experts. To acquire the information related to traceability it is necessary not only to consider the route and different locations of the patient within the system but also the information relating to decision-making and forms such as the nursing evaluation form (admission data, personal data, medical background, evidence of tests, accompanying visitors, medication, administered serums), ICU care form (medication and monitoring of vital signs), etc.

**Figure 1:**
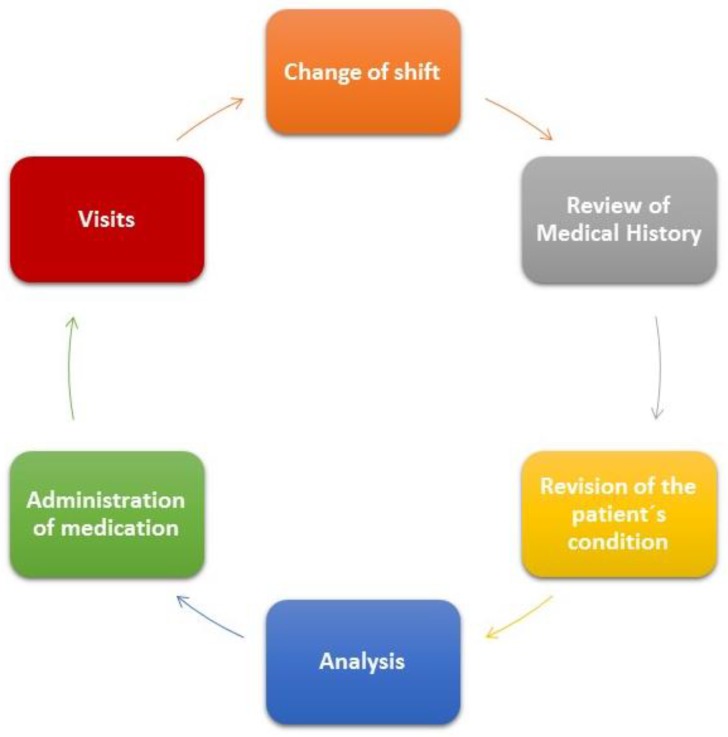
The shift tasks of nursing personnel.

The functioning of these services is accomplished using paper registration and involves various shift changes for the personnel. The first morning shift involves two members of the nursing staff and two nursing assistants. The nursing personnel are involved in the tasks illustrated in Figure 1.

- Shift change: departing health personnel describe to incoming staff the evolution of each one of the patients and inform about new admissions, pending tests to be carried out, etc.
- Revision of clinical history of patients: treatments, diets, tests, etc. for each patient.
- Checking the condition of the patient: absence of pain, sleep quality, etc.
- Extraction of analysis (except for those patients included in seriation of enzymes protocols in which blood is extracted more frequently and not at a specific time) and glucose control.
- Administration of medicine prescribed by the doctor.
- Periodic monitoring of vital signs with a frequency that will depend on the progress and diagnosis of the patient.
- The preparation of medicine for each patient following the consultation of the doctor with the patients to determine progress.
- Reception of family members during the visiting period from 13:00 to 14:00 p.m.

**Figure 2:**
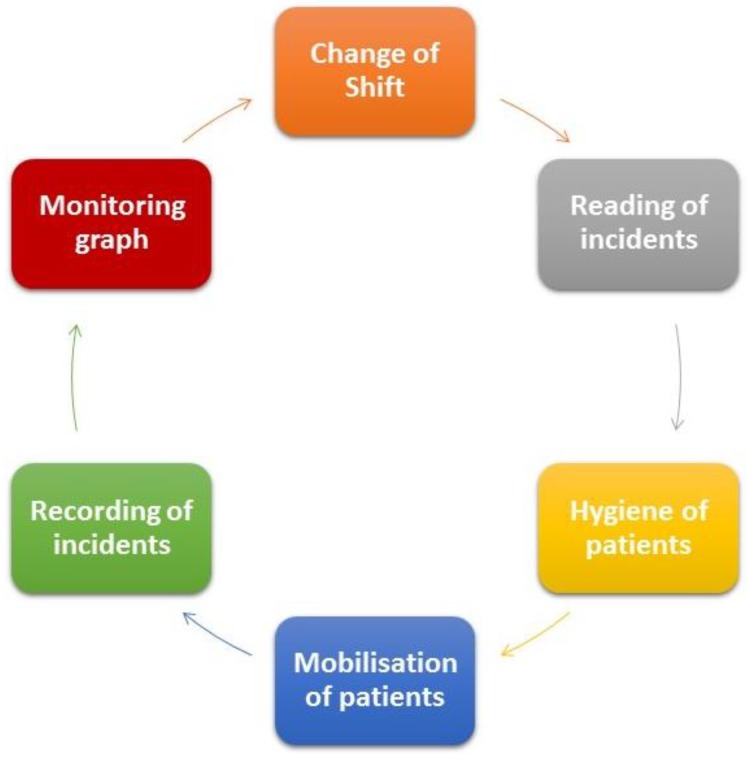
Tasks involved in the first shift of auxiliary nursing staff.

The tasks which auxiliary nurses undertake can be seen in Figure 2:

- Shift changes
- Reading of incidents
- Hygiene of patients
- The changing of posture of individual patients according to the assigned care plans
- Add significant incidents to the incidents register and draw monitoring graph

**Figure 3:**
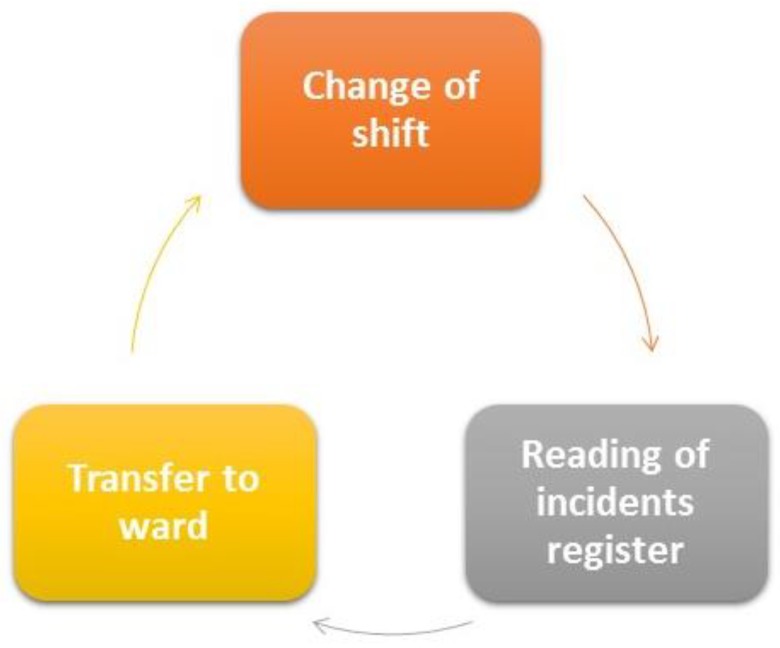
Tasks envolved in the second shift of the auxiliary nurse.

The second shift, which corresponds with the afternoon, involves two nurses and an auxiliary nurse. The nursing personnel carry out the same routines as those referred to in the first shift. However, in the case of nursing auxiliaries, the tasks differ somewhat to those of the first shift and are displayed in Figure 3.

Tasks involved in the second shift of the auxiliary nurse are:

- Shift change
- Read incidents register
- Complete discharge forms for those patients who are being transferred to a ward.

The ICU is one of the most complex areas in the hospital given that its patients tend to receive personalised therapy. As such, it is fundamental that communication between health professionals is standardized, especially in the following situations:

- During shift changes
- Between different care units in the course of transferring a patient from one unit or care service to another

The implementation of computerised medical histories, electronic prescription and discharge systems is recommended which integrate laboratory tests and possible alerts for medication interactions, recommended dose, administration indications etc.

It is important to highlight that this work is considered highly innovative given that it integrates RFID technology into an ICU with quite special characteristics and that traceability is established largely through RFID sources as is described below.

### 3.2. Design

The technical architecture of the developed system comprises two subsystems of hardware and software. Both have been designed to facilitate the flow of information between all the agents involved in the care of the patient.

The software subsystem must correlate with the functional requirements described in the analysis section and the described objectives. Once authenticated by the system, each professional profile will have access to the information in accordance with their professional category and once registered can consult any change in the condition of the patient, almost in real time, as is indicated as follows:

- Healthcare personnel: authenticate, read NFC of the patient, manage the monitor of vital signs, visualise the information of the patient
- Doctor: discharge of the patient, prescribe medication, search for patients based on administered medication lot reference, consult medical history
- Nursing staff: administration of prescribed medication
- Auxiliary nurse: management of care plans

**Figure 4:**
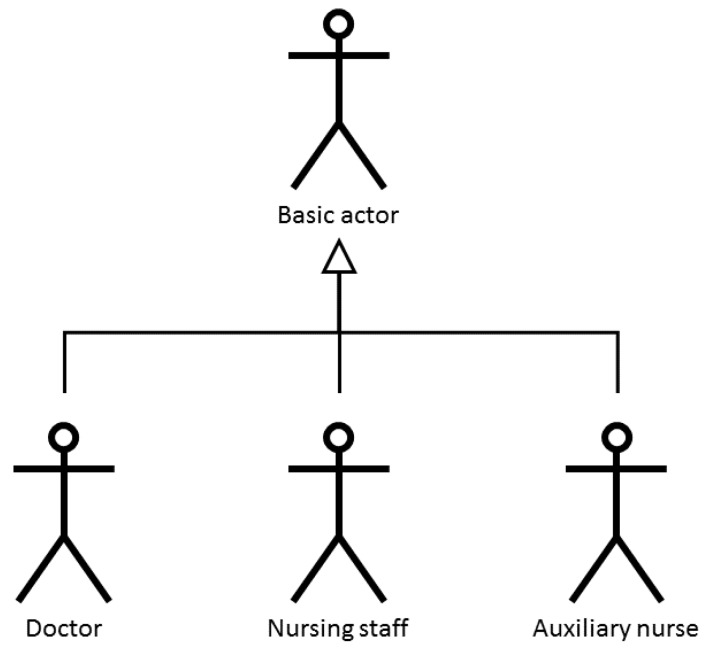
Actors in the subsystem software.

This guarantees safety during the care process of the patient given that no user can carry out clinical practice tasks for which they have not been authorised. Three agents with distinct tasks and privileges have been identified during the design process, (see Figure 4): doctors, nursing personnel and nursing assistants who share tasks at a basic level.

**Figure 5:**
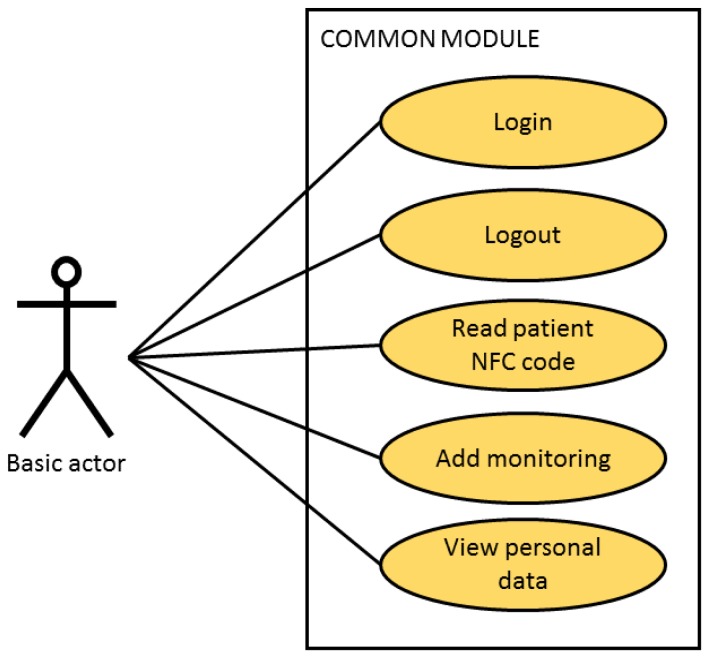
Case of Use of the module common to all users.

To facilitate the design of the software subsystem, it has been divided into distinct modules with each one describing each of the previously described actors. Figure 5 shows the functions available to all of the users. It is important to highlight that everybody has to start the session through the reading of the NFC tag which clearly identifies them in the system.

**Figure 6:**
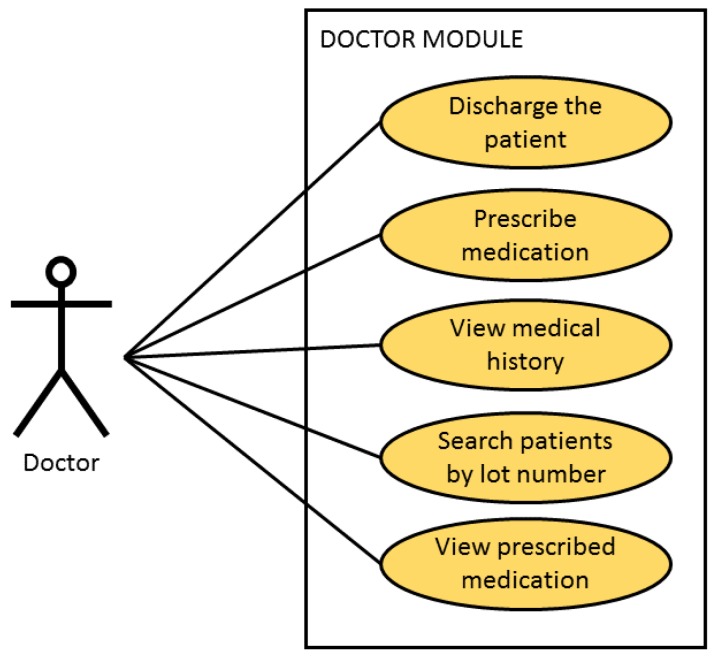
Case of Use of the Doctor module.

The user, once identified, can then read the identity of the admitted patient through the RFID technology and in the case of a patient that has already been assigned, it allows for the management of the monitoring of the patient (the registration of suitable fields: time, temperature, blood and arterial blood pressure). The user can only consult the personal data of the admitted patient for which he/she has been assigned in line with the professional protocol. The following module corresponds with the doctor profile as can be seen in Figure 6.

This module assists the doctor with the discharge of an identified patient following the entry of data for the discharge. Each module includes a series of mandatory fields which the user must complete to continue the task. This module allows for the identification of patients (almost in real time) to whom medication from a specific batch has been administered. This facilitates the management of pharmaceutical alerts and only requires the entry of the name and lot number of the medicine being sought.

**Figure 7:**
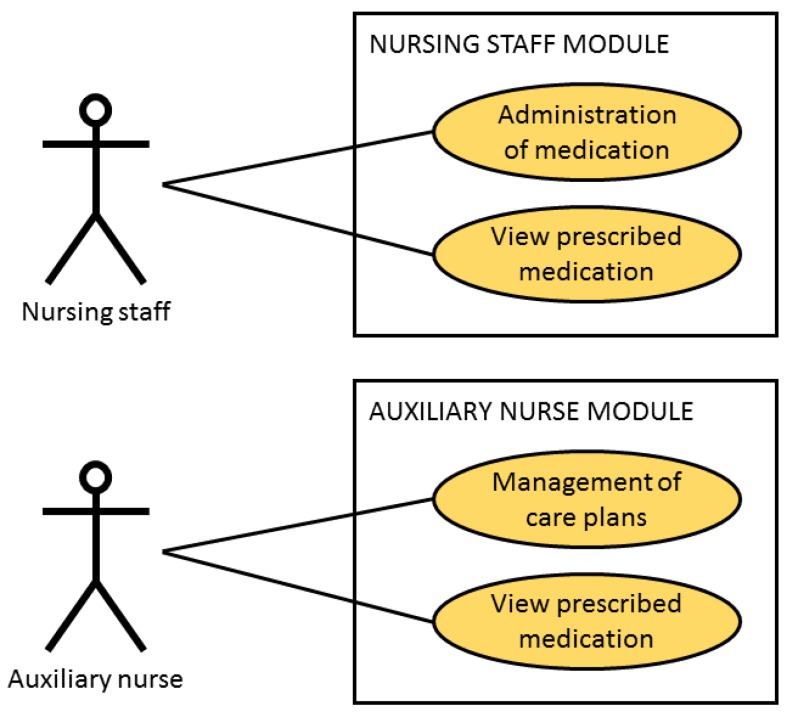
Case of Use of the nursing personnel module and Case of Use of the module by an auxiliary nurse.

The following figure corresponds to the module of the nursing personnel and to the module of the auxiliary nurse as can be seen in Figure 7.

The module of the nursing personnel facilitates the administration of prescribed and pending medication to the patient. The nurse must, firstly, read the NFC tag of the patient and then the NFC tags which identify the medicine to administer. The nursing personnel can consult the list of all the pending and prescribed medication to administer corresponding to their particular shift.

The module of the auxiliary nurse allows the auxiliary nurse to manage (add and modify) the care plans assigned to each patient. This auxiliary nurse can also view the list of medication prescribed and/or administered to the admitted patient.

**Figure 8:**
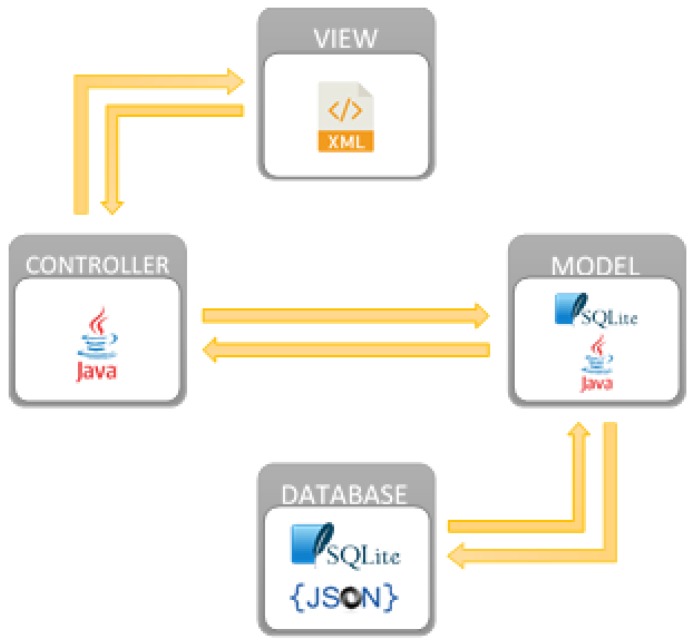
The software architecture of an RFID system designed to obtain traceability.

In the design of the software, the pattern Model-View-Controller has been taken into account as can be seen in Figure 8 in order to add new functionalities and make it easily transferable to other ICUs in other hospital centres.

The effective management of the data (storage, modification and consultation) requires the logical design of the subsystem software. This stage of the design translates the user scenarios into a combination of business objects and services. This is independent of technology and attempts to: refine, organise and detail business solutions in addition to formally defining rules and policies specific to business.

As a result, logic modules are obtained as to what the system has to achieve irrespective of its physical characteristics. The information relating to each of the tables derived from entity-relation is described as follows:

- User: Stores the data common to all users of the application. It comprises name, password and user type (doctor, nurses or auxiliary nurses).
- Category: Stores the value that each user can obtain from the application (doctor, nurses or auxiliary nurses).
- Patient: Stores the personal data of the patients in the application which comprises: clinical history number, name, surname, date of birth, age, nationality, service, bed number and status (discharged or admitted).
- Medication: Stores the data common to the medication that is available within the application. It includes: identifier, lot number, name, dose, routes (oral, intravenous, intramuscular or subcutaneous), expiry date and whether administered or not.
- Monitoring: Stores the common data for monitoring the patient’s status. This includes NHC, time, temperature, blood pressure and pulse.
- Patient discharge: Stores the data related to the discharge of the patient. It comprises the discharge identity, NHC, admittance date, discharge date, recommendations, temperature, blood pressure and pulse.
- Medical history: Stores the data relating to the medical history of the patient. Admission, hospital history and discharge reports. It comprises: NHC, speciality, discharge admission identity, hospital history identity and discharge identity.
- History: Stores the data concerning the hospitalisation of the patient and includes the medication administered. It comprises: medical history, identity, NHC, admission date, medication identity, guidelines, date of start and finish, observations, dosage, routes (oral, intravenous, intramuscular or subcutaneous) and type (whether administered at admission or discharge).
- Alerts: Stores the information relating to patient alerts. Each user is notified of an alert on entering the application. It comprises NHC, medication identity and the reason for alert (it could be because of discharge or a prescription for new medicine).
- Admission: Stores data related to the admission of the patient. It comprises: admission identity, NHC, date of admission, time, origin, diagnosis, blood pressure, temperature, heart rate, respiratory rate, weight and height.
- Administration: Stores the data related to the administration of medication to the patient while in hospital. It comprises: NHC, medication identity, schedule, lot number, date of administration and whether or not administered.

**Figure 9:**
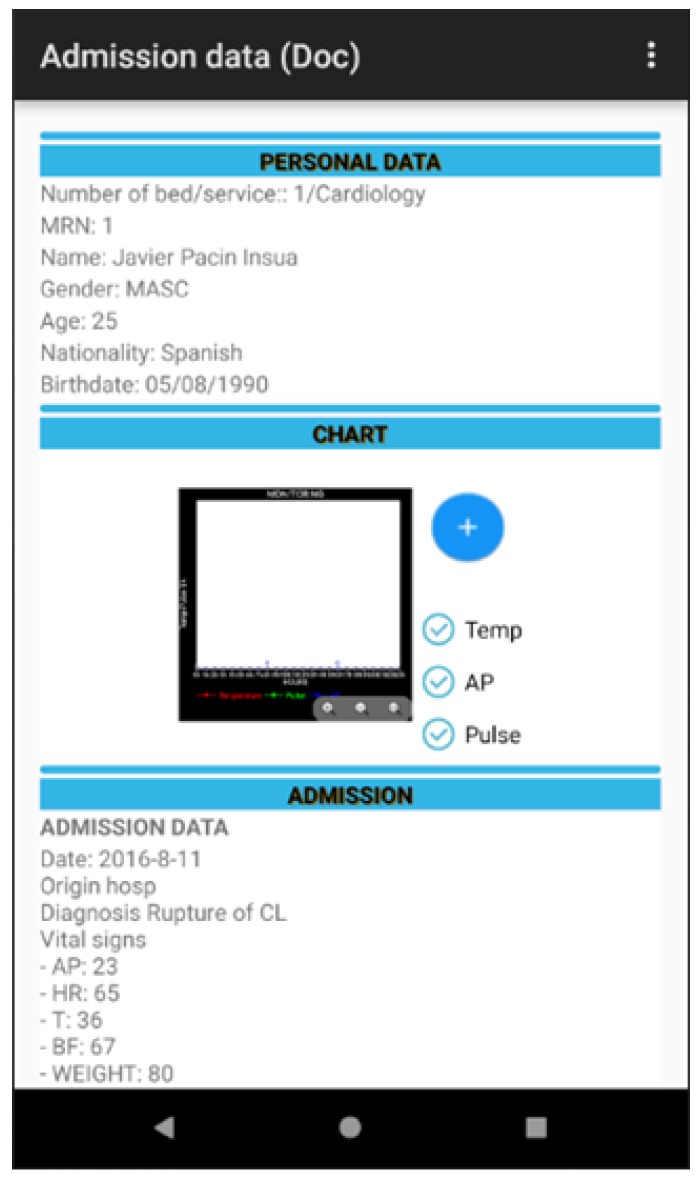
Patient data and vital signs graph monitor.

Another key point in the design of the software is usability given that it is essential that the design includes all the data that health professionals are used to handling. However, this information must appear in the same order and correspond with the chain of tasks that form part of a process in the daily clinical practice of a hospital. At the same time, the software should not be complicated to use and should show maximum and comprehensive information in each one of the screens. This all contributes to the health professional being able to care for patients in a safer and more efficient and rapid way as can be seen in Figure 9.

The hardware subsystem is made up of different RFID systems which is the source of information for the software subsystem. RFID technology allows for the tracking of the patient, medication and the health professionals involved in the care process.

A key point in the design of the RFID system is its operating frequency. This determines the distance at which tags can be read/written and, therefore, the procedure to detect patients, staff or medication identified through RFID.

Following an exhaustive study on the possibilities offered by the different RFID operating frequencies, NFC has been determined to be the most adequate in this case. This is because the aim is to unequivocally identify patients, medicines for administration and the health professionals involved in each of the tasks.

This allows for a comprehensive register to be subsequently available and the tracking of a patient, almost in real time, in his/her route through the service.

NFC adapts to the requisites of this work because patients, most of the time, are to be found at a particular location and readers function at short distances. In addition, patients are normally separated by short distances which favours the functioning of NFC and minimises errors in the reading of patients, medication or nearby personnel that require identification.

Another significant advantage of NFC is that a single mobile device includes all the functionalities required (reading and writing), so that health professionals can manage the RFID system in a rapid, safe and efficient manner.

**Figure 10:**
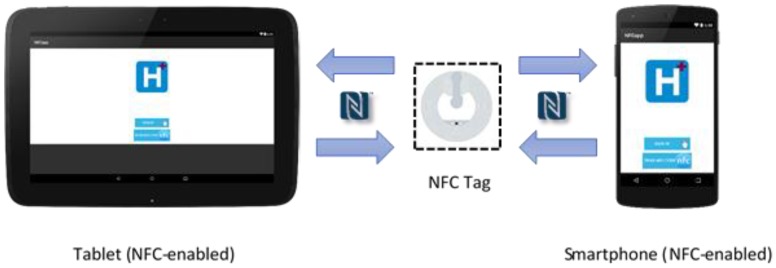
RFID system for tracking in the ICU.

Figure 10 presents a design of the RFID system for traceability in the ICU.

**Figure 11:**
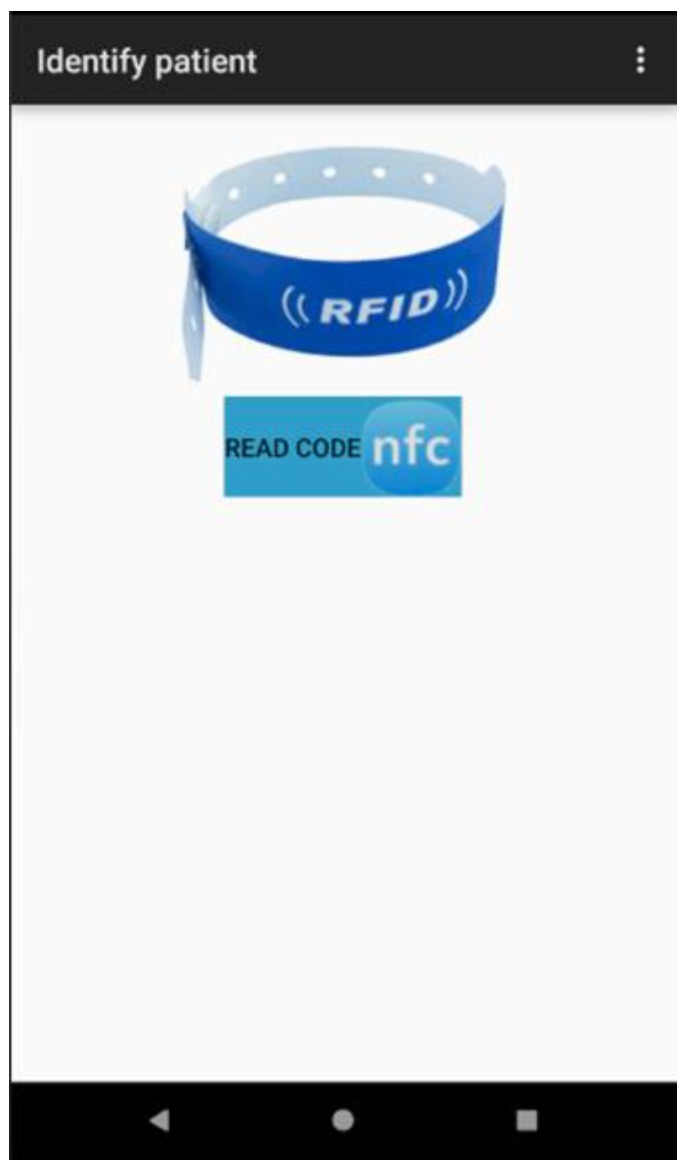
The NFC tag reader in the RFID Tracking System.

In the RFID system, the medication to administer, health personnel and the patient are identified through an NFC tag. This tag must be read by the system (see Figure 11) to obtain the unique identifier (UID) and manage the pending tasks related to the care process of the patient. In the following section, we describe how to carry out the implementation of the system previously described.

### 3.3. Implementation

**Figure 12:**
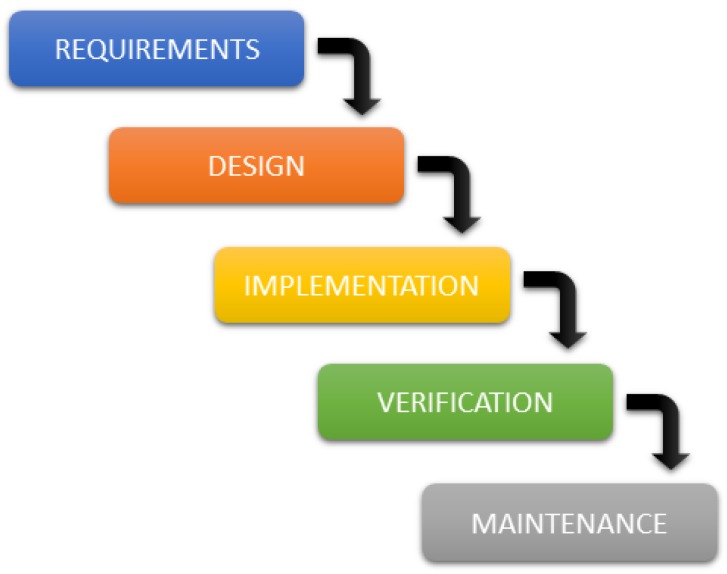
Stages in the Waterfall Cycle.

The implementation of the system has been performed using a waterfall model with feedbacks to previous stages. This is necessary especially when health professionals indicate a new requisite not included at previous stages. The waterfall model represents a linear sequential flow in which software development is conceived as a series of stages executed one after another and follow a flow from top to bottom, like a waterfall (see Figure 12).

The system has been developed for mobile devices using Android, the operating system based on Linux. Android studio has been selected given that it is an official tool and free of cost for Android devices.

Other technologies used in the implementation of this project include:

- JSON is a standard format for the exchange of data. The syntax derives from JavaScript in which the data are name/value pairs.
- JAVA is a general purpose language programme oriented at objects that were specifically designed to allow application developers to execute their programmes on any multiplatform device.
- XML is an extendible mark-up language that defines mark-up languages developed for the World Wide Web Consortium (W3C) which is used to store data in a simple and readable way.
- SQLite is a relational database management system compatible with ACID, contained in a small library and implemented in C.
- Android Test is integrated into Android Studio and simplifies the testing process (based on Junit). It should be pointed out that it can be executed as local unit testing in JVM or as instrumented tests in Android.

It has been a priority that the software employed is responsive in that it can be easily used in any mobile device; the design of an easily usable interface and as similar as possible to the documentation that already exists within the service.

The tracking process requires the unambiguous identification of the patient, medication and health professional through an NFC tag. For this, it was decided to use the Unique Identifier (UID) of the tag to manage the storage of the information corresponding with the database in order to augment the security of the RFID system, given that an external reader cannot access the information relating to the progress of the patient. At the same time, the tags can only be read to avoid any writing on the tags.

**Figure 13:**
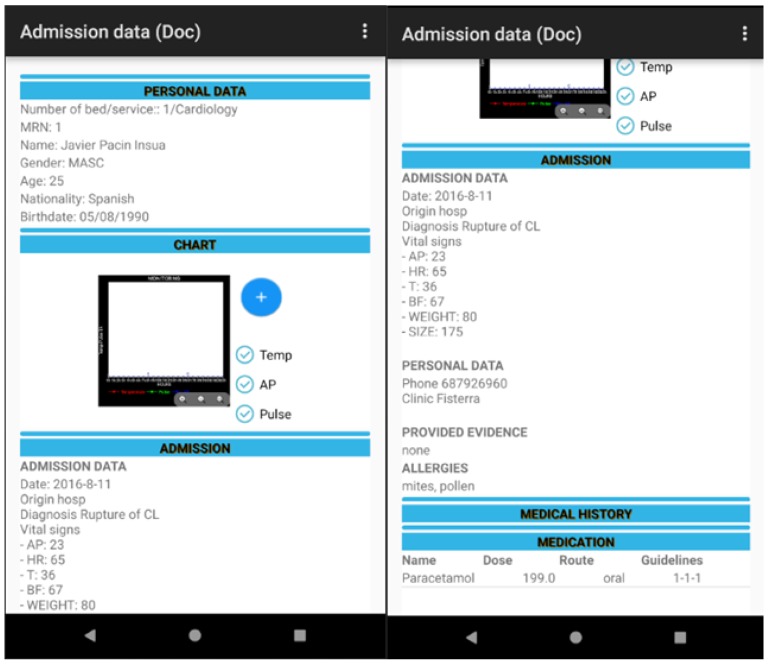
RFID system for tracking in the ICU (doctor).

**Figure 14:**
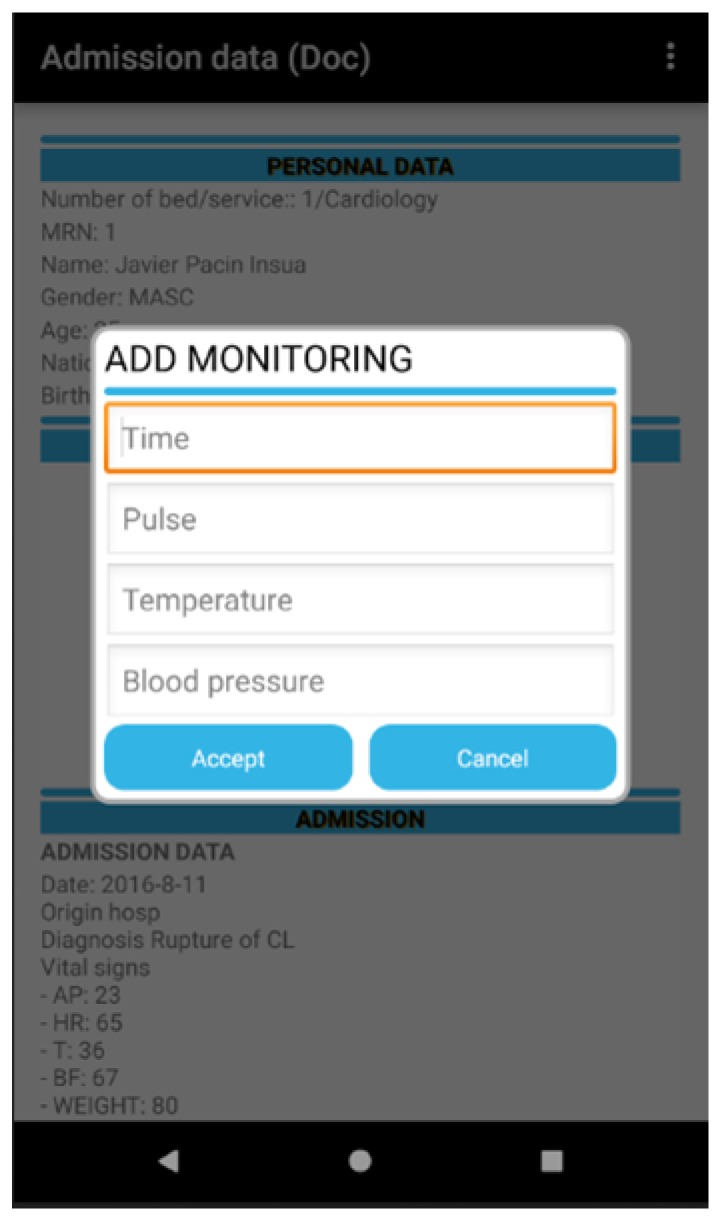
RFID system for tracking in the ICU (nursing personnel).

**Figure 15:**
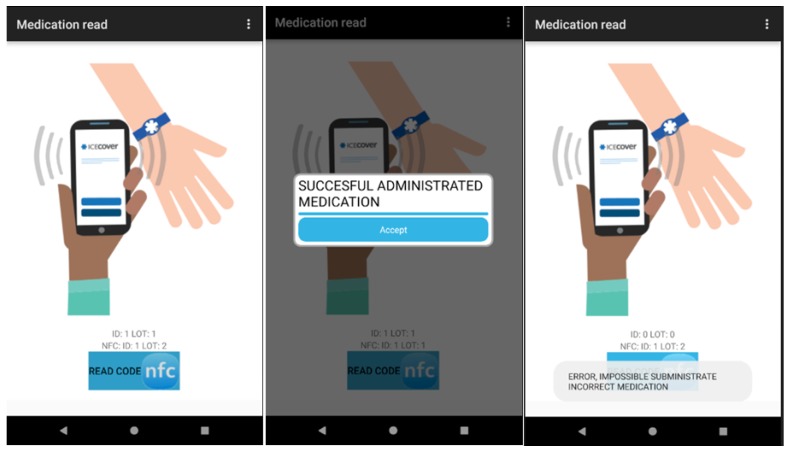
RFID system for tracking in the ICU (nursing personnel).

**Figure 16:**
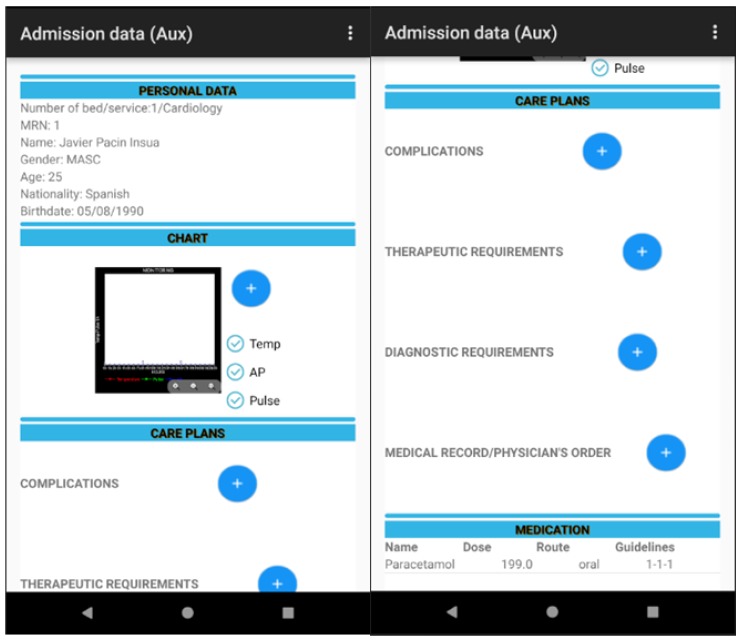
RFID system for tracking in the ICU (auxiliary nursing personnel).

The tool permits the visualisation of the data with respect to the patient: personal, management of the vital signs monitor, admission, prescribed medication (name of medicine, routes, dosage, schedule), etc. in addition to the computerised information between the health team (doctor, nursing and auxiliary staff) including alerts through previously agreed requirements thresholds, the correct administration of medication to the patient, the management of care plans assigned to a particular patient and the identification of patients, in real time, to whom specific lots of medication have been administered to manage pharmaceutical alerts as can be seen in Figure 13, Figure 14, Figure 15 and Figure 16.

The following section describes the tests employed to evaluate the developed system.

### 3.4. Testing and Results

Once the implementation stage has been finalised, it is important to carry out a series of tests to assure the correct functioning of the system. Different types of tests have been conducted:

- Unit tests: These tests validate the correct functioning of each of the functions, that is the NFC readers attached to different materials and the principal modules of the application.
- Integration tests: These tests validate the correct functioning of a combination of integrated functionalities to carry out a more complex task—tasks specific to each health profession profile that require information from various modules.
- System tests: These tests validate the correct global functioning of the implemented system- tasks which involve an overview of the care process of the patient which involves diverse health professional profiles.
- Approval tests: These tests validate the correct functioning of the system on the part of real users, in this case health professionals.

The developed application has been tested in different meetings with at least one representative of each health professional profile to verify and validate its functioning. A range of adjustments, at various levels, have been carried out to arrive at the present state of the application. The users have proposed, in the revision process, changes to the interface to make the change from the traditional paper method to the present application easier. One of the principal modifications, proposed by the health professionals, has been to the interface and the design of how the clinical information of the patient is presented, so that it is clear and simple and adapts to the required standards.

The metrics used to evaluate the system include the following: number of clicks to achieve a task (1–3); number of screens consulted to carry out a task (1–3). In addition, the amount of information required to carry out routine tasks on a single screen, has been checked.

Consequently, the execution time of the tasks always depends on the user but thanks to the design and implementation is much shorter than before (the traditional paper method). The process lasts around 15 s using the application and the handwritten process is clearly longer.

To assure the correct functioning of the system, it has been essential to individually test each of the modules from two perspectives: the tracking of patients, medication and health personnel and on the other, the key elements involved in the care process for each one of the health professionals involved.

Table 1 shows the material necessary to carry out the tests that have been undertaken. The principal problems involved in the reading of certain materials has been resolved with the use of insulating material which reduces interferences with RFID when attached to materials that contain metal or liquids in glass or plastic.

This work has not been developed on a large scale but, if it were, the cost of each acquired unit would notably reduce in price. The larger the number of RFID tags acquired, the less the cost of each unit. The increase in the safety of the patient during the care process in the selected scenario, the ICU, easily justifies the required investment. A period of training for personnel is clearly necessary, during which time it is recommended that both the traditional paper system and developed system coexist so that the number of patients being treated under the new system gradually increases to include all the patients in the service. This period of adaptation allows for improvements in the developed system and a system better adapted to the requirements of the tasks that have to be carried out in daily clinical practice.

The results of the tests have been satisfactory for the clinical personnel who have participated in the evaluation. Performance tests have also been carried out to assure speed and efficiency in the care of the patient during their hospital stay. There have been no reading failures given that with NFC technology, the mobile readers were configured to effect reading with just one physical contact between reader and tag. Some errors were registered: when learning how to handle the reader and with one defective tag. The system is efficient, safe and precise given that the computerization of the data minimizes the possibility for human error during the care process.

An application integrated into the health environment is, in general, different to any other area; not only in relation about that it has to accord with the predefined protocol for those tasks carried out in daily clinical practice but also in the necessity for an interface in which each screen shows the necessary and comprehensive information required by the clinician to attend the patient safely and rapidly. It has not been easy to integrate RFID in this process and computerise the complex tasks involved in this service. For this work, screenshots of the most representative tasks of each health professional have been selected. This work is considered both innovative and cutting edge, not only for the automation of the tasks involved (it contributes to an increase in safety by minimising adverse events and, therefore, the quality of care delivered to the patient) but also for the integration of RFID technology into those tasks.

## 4. Discussion and Conclusions

This work has demonstrated the technical viability of RFID technology in the care process in an ICU with the benefit that only a low cost infrastructure is needed. Given the special characteristics of this unit, the costs in the implementation of an RFID system are clearly justified, and even more so, given that it is essential to increase the safety of the patient with the reduction of adverse events.

The selected operating frequency assures the correct reading of the tags despite personnel, medication and patients situated nearby. The mobile device, as a reader, is comfortable to use and reduces the learning time involved for future users. The system allows for identification, in almost real time, of patients to whom certain lots of medication have been administered, to manage possible pharmaceutical alerts.

Confirmations through NFC are managed through alerts emitted by the application. By reading the NFC tag of the patient and the NFC tag of the medication, the system can confirm whether the medication has been prescribed by the doctor and is pending for administration in this shift or, on the contrary, should not be administered by the nursing personnel because it has not been prescribed or is not scheduled for this particular shift. The administrations that have been attempted but not carried out finally, are not registered although this would constitute interesting future work.

It is possible to integrate this system into other existing tracking systems and it may also be compatible with other identification technologies. Above all, its design has been prepared to be easily transferable to other hospital intensive care units.

## Figures and Tables

**Table 1:** Material acquired for system tests.

Material	Units	Cost/Unit
NFC (NXP NTAG203, ISO 14443 A) passive tags for medication	20	0.10 euros
NFC (NXP NTAG203, ISO 14443 A) passive tags for patients, and health professionals	10	0.10 euros
Android mobile phones with NFC	3	150 euros

## Abbreviations

The following abbreviations are used in this manuscript:

ICU	Intensive Care Unit
RFID	Radio Frequency Identification
UID	Unique Identifier
NFC	Near Field Communication
